# Caregiver Daily Reporting of Symptoms in Autism Spectrum Disorder: Observational Study Using Web and Mobile Apps

**DOI:** 10.2196/11365

**Published:** 2019-03-26

**Authors:** Abigail Bangerter, Nikolay V Manyakov, David Lewin, Matthew Boice, Andrew Skalkin, Shyla Jagannatha, Meenakshi Chatterjee, Geraldine Dawson, Matthew S Goodwin, Robert Hendren, Bennett Leventhal, Frederick Shic, Seth Ness, Gahan Pandina

**Affiliations:** 1 Neuroscience Therapeutic Area Janssen Research & Development, LLC Titusville, NJ United States; 2 Computational Biology, Discovery Sciences Janssen Research & Development Beerse Belgium; 3 Clinical Biostatistics Janssen Research & Development, LLC Titusville, NJ United States; 4 Informatics Janssen Research & Development, LLC Spring House, PA United States; 5 Statistical Decision Sciences Janssen Research & Development, LLC Titusville, NJ United States; 6 Computational Biology, Discovery Sciences Janssen Research & Development, LLC Spring House, PA United States; 7 Duke Center for Autism and Brain Development Department of Psychiatry and Behavioral Sciences Duke University School of Medicine Durham, NC United States; 8 Department of Health Sciences Northeastern University Boston, MA United States; 9 Department of Psychiatry School of Medicine University of California, San Francisco San Francisco, CA United States; 10 Center for Child Health, Behavior and Development Seattle Children’s Research Institute Seattle, WA United States

**Keywords:** autism spectrum disorder, ecological momentary assessment, symptom assessment, mobile app, mHealth, affect, patient reported outcome measures

## Abstract

**Background:**

Currently, no medications are approved to treat core symptoms of autism spectrum disorder (ASD). One barrier to ASD medication development is the lack of validated outcome measures able to detect symptom change. Current ASD interventions are often evaluated using retrospective caregiver reports that describe general clinical presentation but often require recall of specific behaviors weeks after they occur, potentially reducing accuracy of the ratings. My JAKE, a mobile and Web-based mobile health (mHealth) app that is part of the Janssen Autism Knowledge Engine—a dynamically updated clinical research system—was designed to help caregivers of individuals with ASD to continuously log symptoms, record treatments, and track progress, to mitigate difficulties associated with retrospective reporting.

**Objective:**

My JAKE was deployed in an exploratory, noninterventional clinical trial to evaluate its utility and acceptability to monitor clinical outcomes in ASD. Hypotheses regarding relationships among daily tracking of symptoms, behavior, and retrospective caregiver reports were tested.

**Methods:**

Caregivers of individuals with ASD aged 6 years to adults (N=144) used the My JAKE app to make daily reports on their child’s sleep quality, affect, and other self-selected specific behaviors across the 8- to 10-week observational study. The results were compared with commonly used paper-and-pencil scales acquired over a concurrent period at regular 4-week intervals.

**Results:**

Caregiver reporting of behaviors in real time was successfully captured by My JAKE. On average, caregivers made reports 2-3 days per week across the study period. Caregivers were positive about their use of the system, with over 50% indicating that they would like to use My JAKE to track behavior outside of a clinical trial. More positive average daily reporting of overall type of day was correlated with 4 weekly reports of lower caregiver burden made at 4-week intervals (*r*=–0.27, *P*=.006, n=88) and with ASD symptoms (*r*=–0.42, *P*<.001, n=112).

**Conclusions:**

My JAKE reporting aligned with retrospective Web-based or paper-and-pencil scales. Use of mHealth apps, such as My JAKE, has the potential to increase the validity and accuracy of caregiver-reported outcomes and could be a useful way of identifying early changes in response to intervention. Such systems may also assist caregivers in tracking symptoms and behavior outside of a clinical trial, help with personalized goal setting, and monitoring of progress, which could collectively improve understanding of and quality of life for individuals with ASD and their families.

**Trial Registration:**

ClinicalTrials.gov NCT02668991; https://clinicaltrials.gov/ct2/show/NCT02668991

## Introduction

### Background

Autism spectrum disorder (ASD) is a complex neurodevelopmental disorder with a prevalence rate of approximately 1% that is characterized by social communication impairments and restricted, repetitive patterns of behavior [1,2]. There are no approved medications for the core symptoms of ASD, which is in part related to a lack of sensitive outcome measures available for use in clinical trials evaluating potential medications [3,4]. For example, Loth et al 2016 report the failure to detect efficacy in compounds that have shown preclinical promise. The heterogeneity of the ASD population and difficulties with detection of short-term changes have led to recent attempts to develop more sensitive measures including biomarkers [5-7] and novel caregiver rating scales [8,9].

ASD interventions are frequently evaluated using caregiver-reported measures administered during study visits [10]. Two common caregiver measurements of behavior change in ASD are the Social Responsiveness Scale-2 (SRS-2), which measures symptoms associated with ASD [11], and the Aberrant Behavior Checklist (ABC) [3], which measures general behaviors. These surveys require recall of specific behaviors over periods of time, up to 6 months in the case of the SRS-2, which can reduce accuracy of ratings. For example, it was found that caregivers remembered ASD symptoms as being worse in the past compared with current reporting [12]. In addition to problems with retrospective recall, caregivers’ reports can be subject to bias on the basis of context [13]. For example, completing a scale in a clinic context may yield different responses than those provided in settings where the behavior more typically occurs [14]. A respondent’s mood during recall can also influence responses [15]. Finally, measures completed retrospectively are commonly administered before and after an intervention, not during the intervention period, which may limit the ability to assess stability or dynamic change over time and impact detection of potential causal relationships [8,9,16]. Shorter recall periods on symptom reporting scales, for example, the Autism Impact Measure [9] (weeks) and the Autism Behavior Inventory (ABI) [8] (1 week) address this to a certain degree, but these scales could still be influenced by most recent behaviors not necessarily representative of the reporting period.

The advent of mobile health (mHealth) technologies has enabled the development of viable alternatives to retrospective reporting that can facilitate naturalistic logging and recording of behaviors in real or near real time [17,18]. Monitoring of ASD symptoms in a naturalistic setting has the potential to improve reporting validity, leading to increased insight into the condition. Moreover, reporting on symptoms in real time holds the potential to improve accuracy and reliability by reducing episodic memory delays and recall bias [19]. In addition, related to the development of biosensor technologies, real-time observation and reports captured by mHealth have the ability to be used in combination with biometric data to enhance the understanding of the relationship of measures such as actigraphy to classify patterns, symptoms, and behaviors of ASD [20-25].

mHealth approaches could also prove useful in recording transitory factors that reflect or impact mental or behavioral states, such as sleep [26] and caregiver affect [27], items that are more likely to change in the short term. In particular, individuals with ASD have a higher level of reported sleep [28] and psychiatric symptoms, primarily anxiety and mood disorders [29], than the general population; increased anxiety [30-32] and sleep problems [33] are associated with an increase in the severity of the core symptoms of ASD. In addition, a bidirectional relationship has been reported between sleep and internalizing and externalizing behaviors in ASD, as well as a relationship between child sleep and caregiver stress [34-37]. Dynamic measures of these changing states in ASD could lead to better understanding of their effects on core ASD symptoms and help identify more personalized and efficacious interventions.

Paper diaries have been used in clinical trials to obtain information from participants on a daily basis. However, 1 major issue with these approaches, which is prevented with electronic reporting systems, is the reliability of the timing of the report. In 2002, Hufford et al compared the use of paper diaries with electronic reporting and found that although the completion rate was the same at 90%, only 11% of the paper diaries were completed in real time despite what participants reported about their compliance [38].

There is also a need for more idiographic approaches than those afforded by generic scales when considering outcomes that might improve quality of life for patients and caregivers [39,40]. Specific symptoms of most concern to caregivers may not be obvious within change scores of multi-symptom scales, and caregiver-reported responsiveness to treatment may not be detected with these measures [41-43]. Assessments can be enriched by focusing on identifying and monitoring change in problems that caregivers identify as important [44]. As mHealth apps offer the potential for deep personalization, they are well-placed to provide opportunities for tracking real-world, meaningful outcomes.

Although the use of apps to monitor and track health data is expanding [45-47], adoption of e-tech into clinical trials has been slow [48]. Within the field of ASD, there are limited publications regarding user experiences, including opinions about a wide range of features and technologies that mobile apps are capable of monitoring and tracking [49]. Although there are examples of mHealth apps for ecological momentary assessment (EMA) self-reporting in high functioning adolescents with ASD [50-52], to our knowledge, no studies of regular daily or real-time reporting by caregivers of individuals with ASD have been reported. Caregiver reporting may be particularly important for understanding concordance between self-report and caregiver report in ASD and for tracking behavior in younger children or individuals with greater intellectual impairment that might limit the ability to self-report.

### Aims

To address ASD symptom monitoring and tracking needs, the Janssen Autism Knowledge Engine (JAKE), a dynamically-updated clinical research system, was created to provide quantifiable and reproducible measures for use in assessing treatment outcomes, potentially including detection of change in ASD symptoms and behaviors and ASD subgroup identification.

A comprehensive pilot version of JAKE was developed that considered caregiver feedback from focus groups, review of symptoms and symptom descriptions from expert autism clinicians and beta testing [7]. My JAKE is 1 component of the JAKE system and is a Web and mobile app for use by caregivers and clinicians to log symptoms, record treatments, track progress, and gather detailed medical information. Included in My JAKE is the ABI, a Web-based, caregiver-rated scale for assessing ASD core diagnostic symptoms and associated behavior, designed specifically to provide robust and sensitive outcome measures for ASD clinical trials and other interventional studies [8].

One aim of this study was to test the utility of My JAKE as used by caregivers of individuals with ASD who participated in a noninterventional, observational study for a duration of 8 to 10 weeks. In addition, daily ratings of behaviors were compared with the ABI core and associated symptoms of ASD and periodic, retrospectively-collected paper rating scales. Though there was an exploratory component of the study, prespecified hypotheses were created, and they are shown in Table 1.

**Table 1:** Correlation hypotheses of overall type of day, sleep, and mood with autism spectrum disorder (ASD) symptoms and behaviors.

Measure	Hypothesis
Overall type of day	Higher average reports of “overall type of day” will correlate negatively with increased symptoms and behaviors: Core ASD symptoms, challenging behavior, mental health, measured on the ABI^a^ Higher average reports of “overall type of day” will correlate negatively with increased reported caregiver burden on the Zarit Burden Inventory
Sleep	Daily reported sleep quality will correlate negatively with sleep problems reported on the ABI
Mood	Average valence reported in the mood report will correlate negatively with symptoms and behaviors reported by caregivers: ABI and ABC^b^; Average arousal reported in the mood report will correlate negatively with symptoms and behaviors reported by caregivers: ABI and ABC Percentage of reports in each quadrant during the course of the study will correlate with symptoms and behaviors: Quadrant 1 (positive valence, positive arousal) negative correlation with behavior; Quadrant 2 (negative valence, positive arousal) positive correlation with behavior; Quadrant 3 (negative valence, negative arousal) positive correlation with behavior; Quadrant 4 (positive valence, negative arousal) no prediction made

^a^ABI: Autism Behavior Inventory.

^b^ABC: Aberrant Behavior Checklist.

## Methods

### Materials

#### My JAKE

My JAKE is a Web and mobile app comprising various modules to help caregivers of individuals with ASD and their health care providers log symptoms, record participant activities, medical information and treatments, and track progress and change in behaviors. Self-report by study participants was not supported. My JAKE is accessible through most Web browsers, as well as an app for mobile devices, and it was used throughout the study. Caregivers were encouraged to use their own computer and mobile device to access My JAKE. My JAKE includes the following elements.

##### Home Page

**Figure 1:**
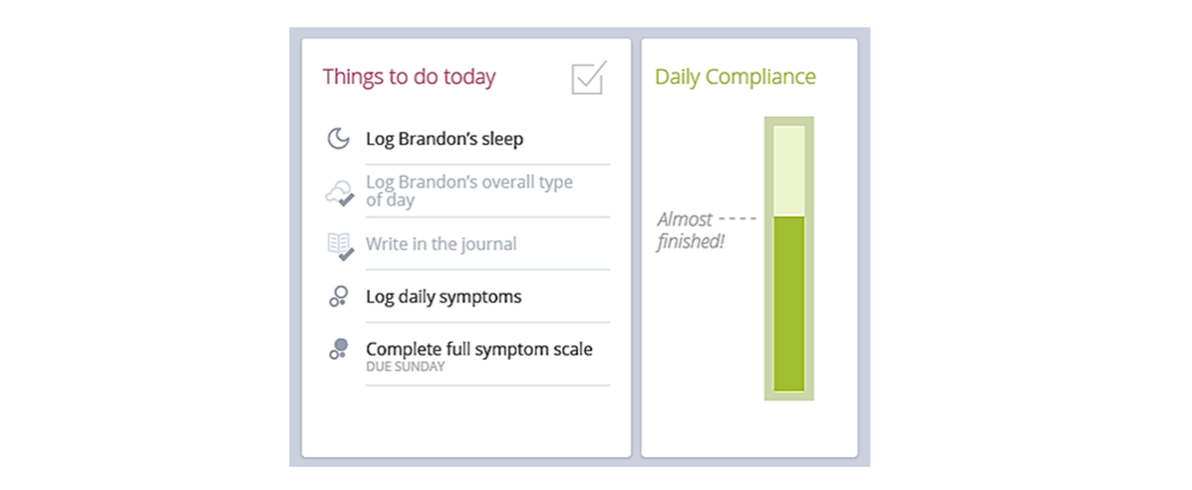
Example of the “to do list” and progress completion apps.

A dashboard on the home page provides an overview of the participant’s appointments, progress, and notifications of My JAKE sections due to be completed (Figure 1). When caregivers log in to the app or the Web version of My JAKE, they will see the “things to do” list, with reminders of what needs to be completed that day.

An external pop up reminder appears outside the app at 8 pm every evening. Clicking on the pop up will take the caregivers to the home page from where they can complete the tasks that had not already been completed for that day. The progress in completing the daily tasks is recorded in the green bar (Figure 1).

##### Medical/Developmental History

A comprehensive medical and developmental history was completed by caregivers during the screening phase of the study. Certain sections, such as developmental milestones, could be completed throughout the study.

##### The Autism Behavior Inventory

**Figure 2:**
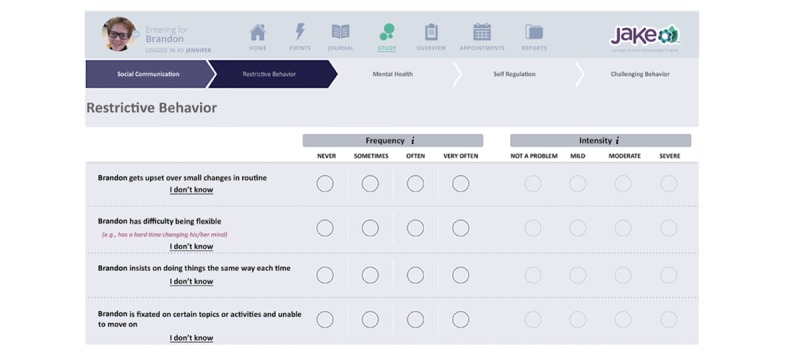
Sample Web-based Aberrant Behavior Checklist to capture autism spectrum disorder symptoms.

The ABI included a series of 73 items related to core and associated symptoms of ASD (Figure 2) [39]. The ABI was completed by the primary caregiver at baseline, midpoint (week 4), and endpoint. Specifically, caregivers are asked to report on the behavior observed in the past week.

##### The Daily Tracker

**Figure 3:**
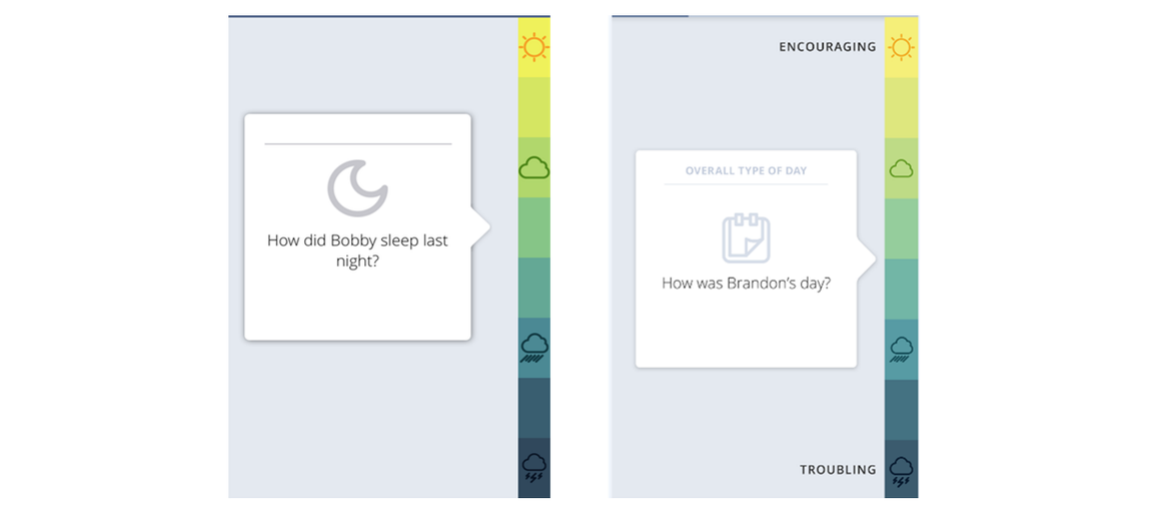
My JAKE Daily Tracker mobile interface: Sleep and overall type of day.

The Daily Tracker was used to measure sleep and overall type of day (Figure 3). It comprised several questions answered by the caregivers of ASD participants. In this study, all caregivers were asked in the morning “How was (participant name)’s sleep last night?” (Sleep) and in the evening “How was (participant name)’s day?” (Overall type of day). Caregivers could choose up to 3 additional behaviors to track. Questions could be answered by dragging a card along an 8-point scale ranging from “troubling” to “encouraging,” either in the My JAKE mobile app or through a Web browser. Picture representation of “good weather” and “bad weather” depicted extremes of the scale, and these icons were selected following extensive user testing with caregivers [7]. Text labels “troubling or encouraging” appear when the user begins to move the card on the screen (shown on right hand side image, Figure 3).

Responses to these questions were tracked and displayed over a 2-week period on the website home page to provide feedback to caregivers and increase engagement in the study.

##### Mood Report

**Figure 4:**
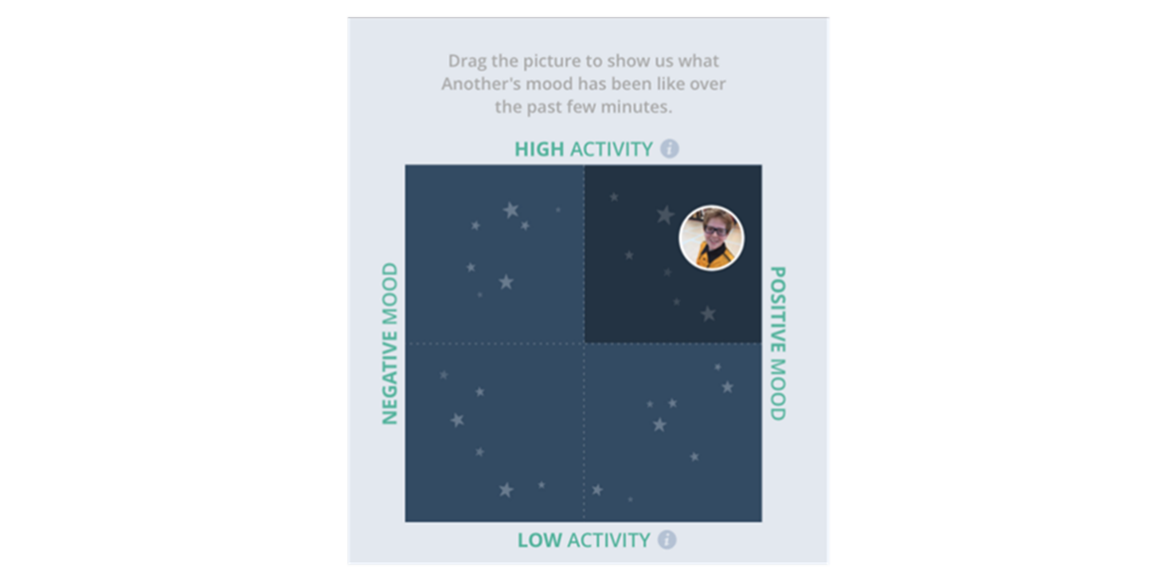
Revised “circumplex” model of affect for the My JAKE mobile app.

The circumplex model of affect has a long and widespread history measuring affective or emotional states, including in those with ASD [53,54]. A digital, square version of the classical affective circumplex was created for the My JAKE mobile app for this study (Figure 4). The horizontal axis represents valence, termed “mood,” and the vertical axis represents arousal, termed “activity.” The model was divided into “Quadrants” of activity and mood relationships. Caregivers were instructed to move the icon representing the participant to a location on the screen that best captured the participant mood at that moment. Caregivers were asked to do this twice a day but could use as many times as desired.

##### Journal and Event Tracker

Caregivers were able to provide ad hoc free-text entries when tracking both positive and negative events as they occurred, and they were able to track all items of interest, including sleep and diet, on a daily basis. A list of common events for ASD, includes repetitive behaviors and tantrums, allowed real-time and retrospective reporting of specific behavior, which would receive a time stamp, enabling comparison with actigraphy and other biosensor data.

##### Therapy Tracker

This module allowed tracking of participants’ medical treatments or therapies using a calendar-like interface. Caregivers could also optionally export created appointments to a calendaring system of their own choice.

##### Connection to Microsoft Health Vault

Microsoft HealthVault [55]—a publicly available, Class 1 electronic personal health record system—was used as the storage mechanism for all data created and accessed by My JAKE. All caregivers were required to create an account in Health Vault to enable registration with My JAKE. The use of HealthVault permitted caregivers to own and control their dependent’s study data, even after the study ended. In addition, caregivers controlled who, as well as which apps, could access their HealthVault account at any time. As an optional service to reduce caregiver burden, Zweena Health, an external company, entered select medical and developmental history data on behalf of participants into the Microsoft HealthVault account, both directly and through My JAKE.

##### Standard Instruments for Measuring Change

Caregiver reported rating scales were used to assess ASD symptom change over time. Scales were selected on the basis of their previous use in clinical trials for ASD or recommendations in reviews of scales for use in measuring change in ASD core and associated behavior [56-58].

The ABC, a 58-item behavior rating scale, was used to measure behavior problems across the following 5 subscales: irritability, lethargy (social withdrawal), stereotypy, hyperactivity, and inappropriate speech. Items are rated on a 4-point Likert scale—ranging from 0 (not at all a problem) to 3 (the problem is severe in degree)—with higher scores indicating more severe problems. The ABC has recently been validated for use in ASD [59,60].

The Zarit Burden Interview—Short Version (ZBI) [61]—is a scale with 22 items designed to assess psychological burden experienced by caregivers. Items ask how the caregivers feel, and responses range from 0 to 4 (never to nearly always). The ZBI has been used to assess burden among caregivers of individuals with ASD [62-68]. Using the ZBI, caregivers of individuals with ASD have been found to experience greater burden of care than other caregivers [63] and burden was related to unmet need in young people with ASD, for example, depression, anxiety, and inappropriate behavior [61,69].

##### The Social Responsiveness Scale 2

Social Responsiveness Scale 2 (SRS-2) identifies presence and severity of social impairment because of ASD. It contains 65 items intended to assess social communication and restricted and repetitive behaviors. A total of 3 forms are available, dependent on the age of the individual with ASD [11].

#### Participants

The study enrolled males and females aged ≥6 years, between 6.0 and 54.0 years of age, with a confirmed diagnosis of ASD on the basis of Diagnostic and Statistical Manual of Mental Disorders criteria. In addition, participants were required to meet research-determined cut-offs identifying a potential diagnosis of ASD according to the Autism Diagnostic Observation Schedule (ADOS-2). Participants were permitted to receive behavioral and/or pharmacologic interventions for ASD and comorbid disorders during the study, but this was not required. However, study inclusion did require participants to live with a caregiver or if not, to spend at least 3 hours a day for at least 4 days each week or at least 3 weekends a month with a caregiver.

Institutional Review Boards approved the study protocol and its amendments. The study was conducted in accordance with the ethical principles that have their origin in the Declaration of Helsinki, consistent with Good Clinical Practices and applicable regulatory requirements. Participants, their caregivers (for participants <18 years old) or legally authorized representatives provided written informed consent before participating in the study. The study is registered at ClinicalTrials.gov (NCT02668991).

#### Study Design

This prospective, noninterventional study was conducted from July 06, 2015 to October 14, 2016 at 9 study sites in the United States. The study comprised a 14-day screening phase followed by an 8- to 10-week data-collection phase. Throughout the study, data were collected via My JAKE and additional caregiver report scales were completed on paper.

#### Procedure

Individuals with ASD and their caregivers were recruited by sites to participate in the study, which involved a screening visit and 3 additional site visits. Participants received a stipend of US $80 to $100 for attendance at each site visit, which lasted around 3 hours. No additional incentives were given for use of My JAKE between visits.

Caregivers were registered to use My JAKE during the baseline visit at the site. The site staff demonstrated the My JAKE app and described requirements for study completion. Caregivers were also provided with a “quick guide” explaining the system and when and how to use it.

The My JAKE home page included an interactive “to do list,” which presented and linked required components (Figure 2). Once a required assessment or measure within My JAKE was completed, it was “grayed out” on the list, and progress toward daily completion was updated. A pop-up reminder outside the app appeared daily at 8 pm. After first completing the ABI at the baseline visit, caregivers could select 3 specific behaviors from the ABI item list for regular tracking through the duration of the study. These formed a part of the daily tracker, along with the additional “overall type of day” question. These questions were available for the caregiver to respond from 6 pm until 12 am. The sleep tracker appeared at 5 am each day and a request to complete the mood tracker appeared twice during the day on the “to do list.” Caregivers were free to use other components of the app, for example, journal, events tracker, in whichever way they wanted.

Site staff members were able to monitor the caregiver’s usage of the app through a clinical dashboard and they had 2 follow up calls (week 1 and week 6) with participants and caregivers at the site visit. Reminders for caregivers to use the app were given at the 4-week midpoint visit, as necessary.

#### Exit Interview

Caregivers were asked to provide final feedback on their experience with My JAKE in an exit interview and by completing a 36-question Web-based survey about system functionality. Examples of questions included “Which JAKE components would you like to use outside of a clinical trial?” and “How easy were the following tasks to complete?”

#### Data Analyses

Correlations between My JAKE daily report measures—for example, 0 to 7 (0=troubling, 7=encouraging) rating on overall type of day, mood report—with ABI and scale domains were calculated using Spearman rank correlations controlling for age and gender.

Features of the mood report analyzed were percentage of time in a given Quadrant—1 to 4 (1=top right, 2=top left, 3=bottom left, 4=bottom right; Figure 4)—percentage of positive valence or arousal, average valence or arousal, and variability of valence or arousal.

All features were averaged over the entire duration of the study and compared with the baseline ABI Core ASD symptoms, associated behaviors, mental health (MH), self-regulation, challenging behavior, and the ZBI. Data were included in these analyses when there were 12 or more reports available.

In addition, the features reported the week before midpoint and the week before endpoint were averaged and compared with scales completed for the corresponding time period. Data were included in analyses for weekly features when there were 3 or more reports available.

## Results

### Study Population

#### Participants

A total of 144 participants with a diagnosis of ASD were enrolled; 136 (94.4%) participants completed the study and 8 (5.6%) participants discontinued. The most common reason for discontinuation was self-withdrawal from the study (6; 4.2%). The majority of the ASD study population sample was male (77.8%), consistent with higher male:female ratio in ASD [49]. Mean (SD) age of participants was 14.6 (7.8) years. Mean (SD) ADOS total score of the participants was 7.6 (1.7), and intelligence quotient (IQ) was 99.2 (19.6).

#### Respondents

A majority of caregivers (41%) were between the ages of 41 to 50 years. Mothers made up 79.9% (115/144) of all caregivers. Most caregivers (32.6%; 47/144) had a bachelor’s degree (Table 2).

**Table 2:** Caregiver demographics (N=144).

Characteristic		n (%)
**Age group (years)**		
	Missing	10 (6.9)
	20-30	5 (3.5)
	31-40	41 (28.5)
	41-50	59 (41.0)
	>50	29 (20.1)
**Relationship**		
	Missing	10 (6.9)
	Father	12 (8.3)
	Grandparent	1 (0.7)
	Mother	115 (79.9)
	Other caregiver (nonfamily member)	1 (0.7)
	Other family member	5 (3.5)
**Education level**		
	Missing	14 (9.7)
	Doctorate degree	3 (2.1)
	Professional degree	6 (4.2)
	Master’s degree	23 (16.0)
	Bachelor’s degree	47 (32.6)
	Some college, no degree	41 (28.5)
	High School graduate	9 (6.3)
	Less than high school	1 (0.7)

##### Data Collected

**Figure 5:**
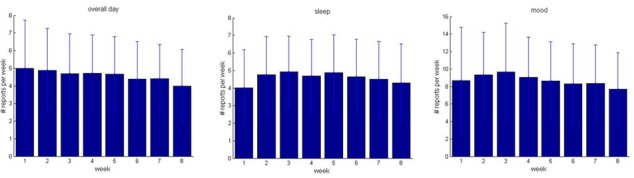
Frequency of caregiver reports over the course of the study, shown as average number of weekly reports with correspondent standard deviations: overall day, sleep, and mood.

Caregivers were asked about overall type of day and sleep once per day and asked to complete the mood reports twice per day (Table 3). The average number of caregiver reports per caregiver, completed over the 8-week study, was highest for mood. A total of 12 caregivers (8%) elected not to use My JAKE during the study. More reports were given for younger participants (<13 years of age) than for older participants (≥13 years of age) over the study period (Table 3). The frequency of caregiver reports obtained by week across the course of the study for overall type of day, sleep, and mood is shown in Figure 5. A linear mixed-effect model with random intercept, having participant as random factor and time (week) as fixed covariate, was used for modeling of weekly number of reports per participant to test whether there were changes in number of weekly reports as a function of time in the study. The number of weekly “overall day” reports per participant significantly decreases at rate 0.025 reports/week (*P*<.001, 95% CI–0.20 to –0.11), and the number of weekly “mood” reports per participant significantly decreases at rate 0.23 reports/week (*P*<.001, 95% CI–0.32 to –0.14). A decrease in reporting of sleep was not significant.

**Figure 6:**
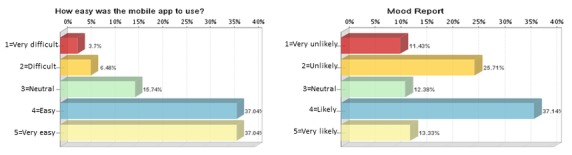
Caregiver rating on the use of the mobile app and the likelihood of using the mood report outside of a clinical trial.

Caregiver ratings were obtained from the exit interview on ease of use and mood report. Most caregivers reported their comfort level using the mobile app as “easy”—37.0 % (40/108)—or “very easy”—37.0% (40/108; Figure 6). Over half of the caregivers responded that they were either likely or very likely to use the mood report outside of clinical trials.

**Table 3:** Average number of caregiver reports given for age subgroups (N=132).

Variable		Mean (SD)
**Mood report**		
	All participants	61.2 (35.62)
	<13 years^a^	67.5 (38.86)
	≥13 years^b^	53.5 (30.54)
**Overall day**		
	All participants	29.8 (35.62)
	<13 years^a^	31.3 (17.62)
	≥13 years^b^	27.9 (16.97)
**Sleep**		
	All participants	28.4 (35.62)
	<13 years^a^	32.1 (19.07)
	≥13 years^b^	23.9 (18.24)

^a^n=72 for <13 years of age.

^b^n=60 for ≥13 years of age.

##### Caregiver Selected Behaviors

Caregivers used the behavior selection component of My JAKE to choose 3 behaviors from the ABI that they wanted to track during the study. The most commonly selected behavior was “sleep problems” (Table 4).

##### Sleep Tracker

Caregivers’ daily reports of sleep displayed a significant negative correlation (*P*<.001) with retrospective reporting on the ABI sleep domain for average rating for the duration of the study and for averaged ratings in the 7 days before midpoint and endpoint visits (Table 5). A negative correlation was expected because a higher score on the ABI sleep symptoms indicates worse symptoms and vice versa.

##### Overall Type of Day

Overall type of day rating scores was averaged across all days in the study and then compared with ABI baseline scale scores for core ASD symptoms, challenging behavior, and MH domains a week after baseline, a week before the week 4 visit, and endpoint. Caregiver rating of overall type of day showed correlations with retrospective caregiver report measures (Table 6). As with sleep measures, a negative correlation was expected with ABI core symptoms, challenging behavior, and MH. In addition, the rating of overall type of day across the duration of the study negatively correlated with caregiver reported burden using the ZBI (n=114, *r*=–0.24, *P*=.005).

##### Mood Report

###### Average Valence

Average valence for the study duration and for the week before midpoint and endpoint was compared with retrospective caregiver reports (Table 7). Average valence was negatively correlated with symptoms and behaviors. In particular, reported negative valence was associated with increased ASD core symptoms (ABI) and increased challenging behaviors across all time points.

**Table 4:** Summary of most commonly selected Autism Behavior Inventory (ABI) daily tracking items.

Rank	Frequency^a^	ABI item selected for tracking
1	24	Has sleep problems
2	20	Acts without thinking
3	19	Has difficulty being flexible
4	18	Is fixated on certain topics or activities and unable to move on
4	18	Worries about things
4	18	Is tense or anxious
7	16	Is irritable and whiny
8	14	Shows inappropriate affection toward unfamiliar people
9	13	Has sensitivities to certain food textures
9	13	Reacts with aggression when he or she is upset or stressed
11	11	Is able to take turns in conversation
11	11	Is anxious in social situations
11	11	Is excessively active
14	10	Directs facial expression toward other people to communicate feelings
14	10	Insists on saying words and phrases over and over

^a^Number of caregivers selecting the item to track.

**Table 5:** Correlation coefficients of daily sleep reports with retrospective reports.

Daily tracker sleep	ABI^a^ sleep
Duration (n=104)	–0.62
7 days before midpoint (n=78)	–0.67
7 days before endpoint (n=78)	–0.63

^a^ABI: Autism Behavior Inventory.

**Table 6:** Overall type of day correlations with Autism Behavior Inventory (ABI) core and other ABI domains, and Zarit Burden Interview (ZBI).

Overall type of day	ABI core symptoms	Challenging behavior	Mental health	ZBI caregiver burden
Study duration (n=112); ZBI (n=114)	–0.42	–0.35	–0.38	–0.24
Midpoint (n=82)	–0.34	–0.42	–0.29	–0.27
Endpoint (n=80)	–0.32	–0.45	–0.26	–0.27

**Table 7:** Correlations of average valence with Autism Behavior Inventory (ABI) core and other domains and autism spectrum disorder (ASD) behaviors.

Timepoint	ABI core ASD symptom scale score	ABI mental health	ABI self-regulation	ABI challenging behavior	ABC^a^ hyperactivity noncompliance	ABC lethargy social withdrawal
Duration (n=111)	–0.26^b^	–0.32^c^	–0.25^b^	–0.29^c^	–0.16	–0.29^c^
Week 4 (n=95)	–0.34^c^	–0.35^c^	–0.21^b^	–0.32^c^	–0.15	–0.25^b^
Endpoint (n=93)	–0.26^b^	–0.12	–0.16	–0.25^b^	–0.06	–0.09

^a^ABC: Aberrant Behavior Checklist.

^b^Significant relationship(*P*<.05) among average valence and ABI core and other domains and ASD behaviors.

^c^Significant relationship (*P*<.001) among average valence and ABI core and other domains and ASD behaviors.

**Table 8:** Correlations of quadrants of activity and mood relationships with Autism Behavior Inventory (ABI) core and other domains, and autism spectrum disorder (ASD) behaviors.

Quadrant	ABI core ASD symptom scale score	ABI mental health	ABI self-regulation	ABI challenging behavior	ABC^a^ hyperactivity noncompliance	ABC lethargy social withdrawal
1	–0.12	–0.30	–0.17	–0.25	–0.07	–0.07
2	0.18	0.26	0.27	0.31	0.21	0.21
3	0.19	0.29	0.18	0.23	0.17	0.17
4	–0.01	0.10	–0.01	0.05	–0.04	–0.04

^a^ABC: Aberrant Behavior Checklist.

###### Average Arousal

Average arousal was also compared for the same time points and no significant correlations were found.

###### Quadrant Reports

The percentage of reports in each quadrant of the mood report across the duration of the study was compared with caregiver reported scales at study baseline (Table 8). Negative correlations were expected between reports in quadrant 1 and increased severity of behavior (Table 1). Those with the fewest reports in quadrant 1 reported challenging behaviors (*r*=–0.25, *P*=.01) and MH (*r*=–0.30, *P*=.001). In contrast, positive correlations were expected among reports in other quadrants. In quadrant 2 (low valence, high arousal), significant positive correlations were found with MH (*r*=0.26, *P*=.006), challenging behavior (*r*=0.31, *P*=.06), and self-regulation (*r*=0.27, *P*=.005). In quadrant 3 (low valence, low arousal) significant positive correlations were found with MH (*r*=0.29, *P*=.003), challenging behavior (*r*=0.23, *P*=.02) and core ASD symptoms (*r*=0.19, *P*=.45). There were no significant correlations for quadrant 4 (high valence, low arousal).

## Discussion

### Principal Findings

To our knowledge, this 8- to 10-week prospective, observational study including caregiver reporting using an mHealth app appears to be the first study of its kind and size in ASD. Caregivers who were using the app, reporting on 4 to 5 days of the week, were able to use My JAKE successfully to report mood, overall type of day, and sleep. There was some decline in reporting over time possibly as caregivers were requested, not mandated, to provide daily reports, but a large proportion of caregivers continued to provide regular reports on their child’s behavior over the course of the 8-week study. Reminders within the system and native to the phone app were limited to once per day, and missed assessments could not be retroactively completed, which meant that daily reports were guaranteed within real time. A few studies have addressed feasibility and compliance with these types of measures, but in a 2-week smoking cessation EMA study, Shiffman et al reported that responsiveness dropped in half of the participants by day 14 [16]. In comparison, a larger proportion of respondents in this study continued to provide daily reports at midpoint (4 weeks) and endpoint. Our data suggest that those caregivers who were still providing reports were doing so at the same rate as when the study began. It was also not unexpected that caregivers of younger children reported more often than caregivers of adolescents and young adults. Due to the exploratory nature of this study, other than the built-in pop-up reminders and midpoint visit, there were no other specific or consistent attempts to increase adherence and participation. However, clinical teams at sites had access to a dashboard, which enabled real-time viewing of data entry points. It would be possible for reports identified as key outcome measures to be more closely monitored and participation to be reinforced through telephone or text reminders by increased initial training on the use of the app. The current feasibility study had many components, including the use of biosensors in the clinic and at home, meaning potentially less focus was on the reporting in the app. In addition, it is expected that in the absence of an intervention, caregiver motivation to monitor and report behaviors will be less. We are currently implementing My JAKE in a 12-week interventional study. This version has some enhanced features for reminders and reduced, more specific requirements for caregivers. It also has an additional training session for parents on the use of the app, with increased support in selecting behaviors of interest to track. Furthermore, in response to parent feedback, interactive features for monitoring and viewing daily reports have been added to the app (whereas they were previously available on the Web). We will observe the influence that these enhancements have on parent reporting.

The use of daily tracking of symptoms by caregivers could be a useful way of identifying early change in response to intervention. Sleep problems were most commonly prioritized by caregivers to track, and 3 items relating to anxiety also appeared in the top 15 items selected by caregivers, confirming research on the prevalence of these cooccurring problems in ASD and the importance of these behaviors to caregivers [70,71]. Other items were consistent with those commonly described by caregivers in clinical settings such as hyperactivity or impulsivity, rigidity, aggression, and problems with social interaction or interpretation [72,73].

Daily measures of behavior showed significant correlations with the ABI and other scales, suggesting that daily electronic tracking of symptoms by caregivers could be a useful way of identifying early change in response to intervention. In particular, the single question regarding “overall type of day” correlated with caregiver report of core symptom severity. Overall type of day also correlated negatively with caregiver report of burden (ZBI), consistent with research on the impact of ASD symptoms and behavior on caregiver strain [74,75], and it has a possibility, with further validation, for use as a global index for caregiver-reported change. It is difficult to address the validity of a new measure, especially in relation to an existing measure [16]. Some concordance is expected, and it was seen in this study, indicating that similar constructs and concepts were being reported by caregivers in both real time and retrospectively. Further triangulation of data is important, for example, use of biosensor actigraphy information alongside daily sleep reports, and consideration of the relationship between physiological changes, detected by biosensors, and parent daily report as well as retrospective reports. This will be a focus of data analysis in our next clinical trial using My JAKE. In addition, we will be investigating the relationship of daily reports with measures of symptom and behavior change from other informants, such as the Clinician Global Impression of Severity [76].

Caregivers reported that they found My JAKE easy-to-use, which was a likely contributor to their willingness and expressions of interest in using the system outside of a clinical trial. In particular, caregivers made use of the mood report, which allowed them quick and easy reporting on 2 dimensions of valence and arousal with a single action. Although valence correlated with ABI domains, arousal did not. The use of quadrants that combined the 2 features is a potentially useful way to measure and report mood, as it takes into account the second dimension of arousal. Further investigation into the relationship of quadrant reports and behavior will be carried out to establish the value of the 2-dimensional mood report for measuring mood in ASD. In addition, variability in mood or caregiver affect as interpreted by a caregiver or observer is a feature of particular interest when caregivers report regularly over a period of time. It should be noted that no anchor words were provided for reference on any part of the mood report, that is, caregivers were left to provide their own definitions while rating valence and arousal. Thus, the scale may be partly self-referential, and may require other analytic methods to objectively characterize scale performance (ie, operationally defining clinically meaningful change).

### Study Limitations

The caregiver burden in this study was considerable. In addition to either filling in or curating a lengthy medical and developmental history, caregivers were expected to use My JAKE to report on their child’s behavior several times per day and this led to missing data. A better sampling strategy, including focus on only a few elements, is currently being implemented in an interventional study to prevent data loss and could contribute to keeping caregiver burden low and quality and utility of data high.

We also observed a differential pattern of reporting that included fewer assessments completed for caregivers of adolescents and adults compared with children. This may be because of a lack of awareness of behaviors, less frequent contact or observation opportunity, or other factors, and it should be taken into consideration when selecting completion requirements in future studies.

Although this was a relatively heterogeneous group of individuals with ASD, it would be helpful to obtain data for participants across the spectrum of severity and IQ to determine whether there are meaningful patterns of differences. This would necessarily require a much larger sample than allowed by this initial study.

Data were generally provided by the primary caregiver. It may be important to have data from other collateral sources such as school or program staff or self-reported data from individuals with ASD who are capable of providing feedback on their own behavior.

Though My JAKE was not used by caregivers for EMA in this study, it does have the capacity to do so with a minor modification. However, the burden of more frequent prompts and reminders for caregivers would need to be considered, in contrast with the need for consistency of many repeated measures over fluctuating periods of time. In addition, caregivers are not always with the child to report behaviors in real time (or the nature of a distracting behavior itself may make reporting difficult). The ability of My JAKE to be used for real-time and retrospective reporting of events increases its flexibility to be used to collect the type of data most relevant to outcomes of interest for a particular intervention while considering what is least burdensome for caregivers. We plan to use the event reporting from our current intervention study to correlate with biosensor data obtained from an actigraphy watch that participants with ASD will wear over the 12-week period. The use of biosensor data will also improve our understanding of the content validity of daily assessments, providing an opportunity for comparison with objective measures alongside the retrospective parent reports.

### Next Steps

My JAKE is currently employed in an interventional clinical study in ASD. Learnings from this study relating to frequency and burden of caregiver reporting, specific instructions to caregivers, and ability of study sites to train and support caregivers in regular use of the app have been used to ensure maintenance of quality and useful data for measuring outcomes. The current version of My JAKE is also suitable for multiple raters, including teachers and other health care professionals, enabling the possibility of additional reporting across a broader range of contexts, settings, and perspectives. Finally, with some adaptations, the app has the potential to allow for self-reporting in more cognitively-able and older individuals with ASD. This would add an important dimension to the assessment of change in symptoms over time.

### Conclusions

To our knowledge, this study represents the first reported implementation of an mHealth app in a large number of caregivers for electronically tracking behaviors in ASD. Caregivers were successful in using My JAKE to report behaviors, and reported behaviors largely agreed with other more traditional retrospective reports. The study findings will aid in selecting specific outcome measures, leading to fewer and more specific reporting requirements from caregivers, with the expected result of reduced caregiver burden, improved data quality, and potentially more sensitive and meaningful outcomes in monitoring behavior of individuals with ASD.

## Abbreviations

ABC: Aberrant Behavior Checklist
ABI: Autism Behavior Inventory
ADOS: Autism Diagnostic Observation Schedule
ASD: autism spectrum disorder
EMA: ecological momentary assessment
IQ: intelligence quotient
JAKE: Janssen Autism Knowledge Engine
MH: mental health
mHealth: mobile health
SRS-2: Social Responsiveness Scale 2
ZBI: Zarit Burden Interview
